# Metabolic Fate of Dietary Glucosinolates and Their Metabolites: A Role for the Microbiome

**DOI:** 10.3389/fnut.2021.748433

**Published:** 2021-09-22

**Authors:** John A. Bouranis, Laura M. Beaver, Emily Ho

**Affiliations:** ^1^Linus Pauling Institute, Oregon State University, Corvallis, OR, United States; ^2^School of Biological and Population Health Sciences, Oregon State University, Corvallis, OR, United States

**Keywords:** bacteria, broccoli sprouts, cruciferous vegetables, glucosinolate, isothiocyanate, microbiome, sulforaphane, sulforaphane nitrile

## Abstract

Robust evidence shows that phytochemicals from cruciferous vegetables, like broccoli, are associated with numerous health benefits. The anti-cancer properties of these foods are attributed to bioactive isothiocyanates (ITCs) and indoles, phytochemicals generated from biological precursor compounds called glucosinolates. ITCs, and particularly sulforaphane (SFN), are of intense interest as they block the initiation, and suppress the progression of cancer, through genetic and epigenetic mechanisms. The efficacy of these compounds is well-demonstrated in cell culture and animal models, however, high levels of inter-individual variation in absorption and excretion of ITCs is a significant barrier to the use of dietary glucosinolates to prevent and treat disease. The source of inter-individual ITC variation has yet to be fully elucidated and the gut microbiome may play a key role. This review highlights evidence that the gut microbiome influences the metabolic fate and activity of ITCs. Human feeding trials have shown inter-individual variations in gut microbiome composition coincides with variations in ITC absorption and excretion, and some bacteria produce ITCs from glucosinolates. Additionally, consumption of cruciferous vegetables can alter the composition of the gut microbiome and shift the physiochemical environment of the gut lumen, influencing the production of phytochemicals. Microbiome and diet induced changes to ITC metabolism may lead to the decrease of cancer fighting phytochemicals such as SFN and increase the production of biologically inert ones like SFN-nitrile. We conclude by offering perspective on the use of novel “omics” technologies to elucidate the interplay of the gut microbiome and ITC formation.

## Introduction

### Cruciferous Vegetables and Cancer Prevention

Cancer is the second leading cause of death in the United States and high cruciferous vegetable consumption has been associated with lower risk of breast, prostate, lung, colorectal, bladder, endometrial, gastric, ovarian, renal, and pancreatic cancer (1–17). Cruciferous vegetable consumption offers a possible cost-effective and appealing non-pharmacological approach to cancer prevention through dietary intervention. Glucosinolates (GLS) are a class of compounds ubiquitously contained in cruciferous vegetables, that when metabolized, have been shown in animal and cell culture models to prevent and suppress cancer formation (18–28). A single type of cruciferous vegetable will contain a wide variety of GLSs, however, one to three are typically present in the greatest abundance (29). Broccoli, Brussels sprouts, bok choy, collard greens, cabbage, cauliflower, Chinese cabbage, kale, kohlrabi, mustard, radish, rutabaga, turnips, swiss chard, watercress, and other cruciferous vegetables are a rich and unique source of GLS (30). A GLS can be metabolized to an isothiocyanate (ITC), indole, or nitrile (31, 32). ITCs and indoles are bioactive and largely considered responsible for the anti-cancer properties of these foods, while nitriles and GLSs are thought to be biologically inert (18, 33). The most heavily studied ITCs and indoles are sulforaphane (SFN) and indole-3-carbinol (I3C), respectively (18–25, 34–38).

SFN and I3C have a plethora of anti-cancer bioactivities that can be grouped into two primary mechanisms, the first of which is “blocking” the initiation of cancer and the second is “inhibiting” the progression of tumor growth and metastasis [reviewed in (39)]. The blocking mechanisms are primarily related to the modulation of Phase I and Phase II drug metabolizing enzymes which prevent the activation of pro-carcinogens and increase the clearance of xenobiotics and carcinogens. I3C increases the transcription of Phase I enzymes while SFN works in a complementary manner to upregulate Phase II enzymes (18–24, 36, 40–47). In addition to these activities, I3C can also alter sex hormone metabolism, playing a role in preventing hormone-sensitive cancers (48, 49). Post-initiation, SFN and I3C can help inhibit the growth of tumors by inducing apoptosis and halting cellular proliferation (19, 21, 24, 50–52). SFN and 3-3′-diindolylmethane, the acid condensation product of I3C, have also been shown to inhibit enzymes that regulate epigenetics whose dysregulation contributes to cancer development (25, 37, 53, 54). For example, in cancer cells, administration of SFN has been shown to decrease the catalytic activity of histone deacetylase while 3-3′-diindolylmethane has been shown to decrease the levels of HDAC proteins, restoring the activity of tumor suppressor genes (25, 34, 37, 38, 53–56). Other ITCs, including allyl isothiocyanate (AITC), benzyl isothiocyanate, and phenethyl isothiocyanate, from cruciferous vegetables have been shown to have anti-cancer properties as well (47, 57, 58). In this review we will primarily focus on GLS metabolism to ITCs, related nitriles, and the role the microbiome may play in their metabolism. Indoles are reviewed by David E. Williams in this same issue.

### Variability in Human Studies: A Case for the Gut Microbiome

While cell culture and animal models present robust evidence supporting the anti-cancer potential of ITCs, human clinical trials examining the efficacy of whole food interventions on cancer prevention targets have shown high levels of inter-individual variation in both the absorption and excretion of ITCs [reviewed in (59)]. The source of this variation is still unknown and is critical to understand as low levels of GLS conversion to ITCs may limit the use and efficacy of diet as a strategy to reduce cancer risk. Inter-individual variation following GLS consumption has been described in both food-based and glucoraphanin (GRP) supplement clinical trials (34, 60–65). In a study where 45 participants received a standardized dose of GLS orally (primarily GRP), ITC conversion rates ranged from 1.1 to 40.7% of the given GLS dose (66). Inter-individual variation of ITC absorption has also been described for other cruciferous vegetables like watercress which highlights that variation in metabolism likely extends to a wide variety of GLS and their bioactive ITCs (67, 68).

Much of the early work that examined sources of ITC inter-individual variation focused on glutathione-S-transferase (GST) polymorphisms, as a major factor modulating effects of ITC metabolism, but studies are inconsistent and equivocal (69–81). Our own group has shown that *Nrf2* KO mice, which lack the ability to induce GSTs, do not manifest differences in SFN metabolite production (82). As an alternative, it has been proposed that differences in individuals' gut microbiomes may contribute to the observed variation through the production of ITC or inert NITs from GLS, which we discuss here in detail. Likewise, we discuss how consumption of cruciferous vegetables may alter the microbiome and in turn influence ITC absorption. The gut microbiome is well-known to play a critical role in the metabolism of other bioactive chemicals obtained from food sources. Research in the field of soy isoflavone metabolism has shown that the gut microbiome not only plays an integral role in the metabolism of isoflavones, but also determines their efficacy (83, 84). Similar responder/non-responder paradigms have been observed with urolithins from pomegranate extract, with three distinct “metabotypes” having been identified and correlated with differential gut microbiota compositions (85). Cumulatively, these findings suggest that a similar paradigm may exist regarding cruciferous vegetables metabolism.

While this review focuses on how the microbiome may contribute to high levels in inter-individual variation in GLS metabolism, other host derived factors that may also affect variation include mastication, digestion, meal composition, body mass, and use of medications [reviewed in (29, 86)]. It is also important to acknowledge that variation in detecting relationships between cruciferous vegetables and cancer prevention at the population level may also be related to variation in GLS concentrations and metabolism in the vegetables including the variety of vegetable, soil type, growing conditions, growing methods, and age of plant at harvest (87–89). Likewise, differences in vegetable processing, cooking methods, and time of day the GLS are consumed also affects the metabolism, absorption and excretion of ITC [reviewed in (66, 90, 91)].

### Glucosinolates and Their Metabolic Products

Biologically important glucosinolates include GRP, glucobrassicin, glucoerucin (GER), glucoiberin (GIB), sinigrin (SNG), progoitrin, glucotropaeolin (GTP), and gluconasturtiin (GNT) (30, 31). GLS are relatively stable in the plant cell and are composed of a thiohydroximate-O-sulfonate group linked to glucose, and an alkyl, aralkyl, or indolyl side chain [reviewed in (92)]. Metabolism of GLS often begins when the raw plant tissue is damaged by cutting, or mastication and the thioglucose bond in the GLS is cleaved by myrosinase (31, 92). Myrosinase is a β-thioglucoside glucohydrolase which is stored in specialized myrosin cells, preventing a reaction between GLS and myrosinase until damage to the plant cell wall occurs (93, 94). Within the active site of myrosinase, a nucleophilic attack by glutamate on the anomeric carbon of the GLS begins the reaction, leading to the aglycone product being released, which then undergoes a Lossen-type rearrangement (94). Ascorbic acid acts as cofactor deprotonating a water molecule which then attacks the anomeric center of the substrate resulting in the release of the bound glucose from the glutamate residue (94). The products from this reaction depends on the conditions [ex. pH and described elsewhere (31, 91)], proteins present (ex. Epithiospecifer protein), type of GLS and results in the formation of ITCs, indoles, nitriles (NITs), epithionitriles, oxazolidine-thiones, and thiocyanates (29, 31, 95, 96). It is critical to note that many GLS obtained *via* diet may not be converted to ITCs because many methods of cooking inactivate myrosinase in the vegetable, and myrosinase-like activity does not occur in mammalian cells (31, 91, 97, 98). Importantly some gastrointestinal microflora have myrosinase-like activity highlighting an important role for the microbiome on GLS metabolism (99–110).

**Figure 1:**
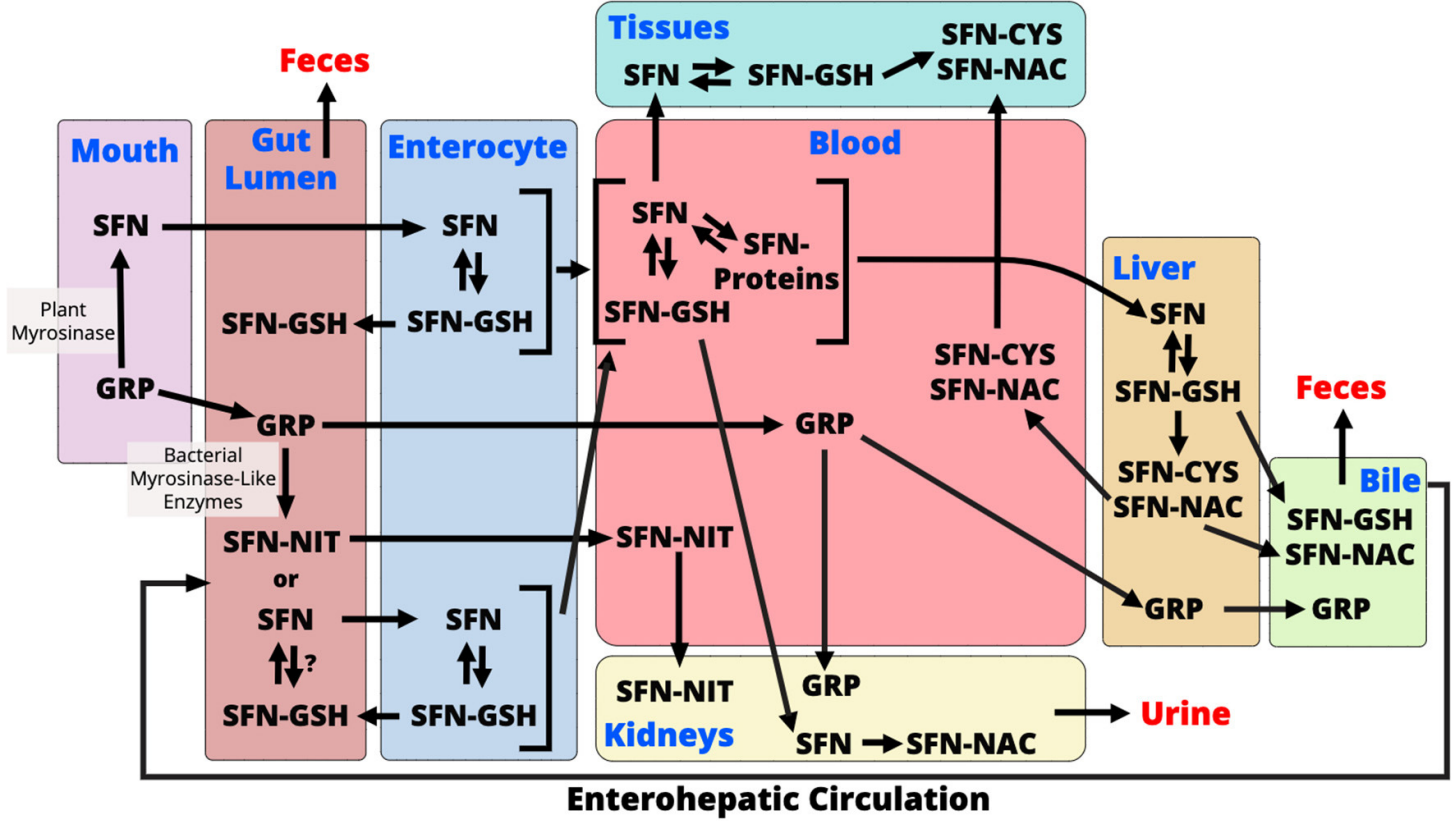
During chewing of raw broccoli sprouts, plant myrosinase converts glucoraphanin (GRP) to sulforaphane (SFN) in the mouth. In the gut lumen, GRP is further converted to either SFN or sulforaphane-nitrile (SFN-NIT) by the gut microbiome. It is unclear if microbes conjugate SFN to glutathione (GSH), however, only free SFN is taken up into enterocytes. Within the enterocyte, SFN-GSH is either excreted back into the gut lumen or enters circulation where it is exists as SFN-GSH or in its free form which conjugates with blood proteins. SFN is transported to either tissues where it exerts its bioactivity or to the liver where it is metabolized *via* the mercapturic acid pathway. Mercapturic acid metabolites, SFN-cysteine (SFN-Cys) and SFN-N-Acetyl-Cysteine (SFN-NAC), from the liver are either exported to the bile for excretion to the feces, or back into the blood to go to tissues. SFN also goes to the kidney where it is converted to SFN-NAC and excreted into the urine. Un-hydrolyzed GRP is either excreted into the feces or absorbed where it is either transported to the kidneys to be excreted in urine or to the liver where it is excreted into bile. GRP that undergoes enterohepatic circulation and is either hydrolyzed in the gut or excreted to the feces. SFN-NIT is absorbed from the gut lumen where it is transported to kidneys for excretion into urine. SFN-NIT metabolism in humans needs further investigation.

ITC have been shown to have a wide range of biological effects, and NITs are considered biologically inactive although this is also controversial (18, 33, 111–113). AITC, allyl nitrile, benzyl isothiocyanate, benzyl nitrile, iberverin, iberverin nitrile, iberin (IBN), iberin nitrile, erucin (ERN), erucin nitrile, phenethyl isothiocyanate, and phenethyl nitrile are ITCs and NITs that are largely studied to date. Here we will discuss these compounds but also focus our discussion around SFN as it is the most well-studied ITC. Like other ITCs, SFN metabolism begins with how the vegetable is prepared. When eaten raw, or lightly cooked such that myrosinase is active, GRP is cleaved to produce SFN (Figure 1). SFN appears to be absorbed into the enterocyte by passive diffusion and the rapid conjugation of SFN to glutathione potentiates this process (114, 115). Intracellular SFN-glutathione is exported from enterocytes back into the gut lumen by membrane bound proteins (115, 116). SFN metabolites exit the enterocyte into circulation where they are predominantly conjugated to glutathione, however, unbound SFN can bind to blood proteins and has been detected in mice and humans (82, 117). SFN is further metabolized in the liver through the mercapturic acid pathway where compounds are first conjugated to glutathione, *via* a reaction catalyzed by GSTs, then further metabolized to cysteinylglycine (CysGly), cysteine (Cys), and N-Acetylcysteine (NAC) conjugates (30, 31). SFN-NAC is the major metabolite detected in urine. In the absence of myrosinase, GRP may be metabolized by the gut microbiome or it can be directly excreted with no biological activity (Figure 1) (91). It is unclear if microbes conjugate SFN and other ITCs to glutathione following GLS hydrolysis, however, SFN must be in its free form in order to enter into the enterocyte (114). Conversion of GRP to SFN-NIT, as opposed to SFN, could lead to a reduction in bioactivity (Figure 1). Endogenous SFN-NIT metabolism is not well-understood although allyl nitrile metabolism has been described (118). SFN-NIT can also be produced by vegetable-derived myrosinase when GRP hydrolysis occurs in the presence of epithiospecifier protein (ESP) (95, 96, 119, 120). Members of the microbiome may also produce SFN-NIT from GRP (Figure 1 and discussed herein).

The bioavailability of ITCs from GLS has been shown to be greatly impacted by processing before ingestion [reviewed in (31, 92, 121)]. When ITCs are given preformed, such as those found in supplements, they are readily absorbed by humans and possess the greatest level of bioavailability. ITCs from raw cruciferous vegetables, while less bioavailable than those from supplements, additionally have a high bioavailability, as much GLS is converted to ITCs by endogenous plant myrosinase in the mouth during chewing or before consumption during the chopping of vegetables (86, 92, 93, 122). Cooking has been shown to greatly decrease bioavailability as heat deactivates endogenous plant myrosinase, thus any conversion of GLS to ITCs would occur in the gut lumen by the gut microbiome (31, 66, 67, 92, 93, 97, 109, 121, 123).

## Microbial Metabolism of Glucosinolates

### GLS Hydrolysis to Bioactive Compounds

This review will focus primarily on ITCs and NITs produced from GLS by members of the gut microbiome because little is known about the extent to which bacteria metabolize GLS to other metabolic endpoints. The most heavily studied of GLS biotransformation by gut microbes is the hydrolysis of GLS to bioactive ITCs. It is well-known that plant myrosinase generates ITCs from GLS, however, heat-treatment, such as during cooking, deactivates plant myrosinase, limiting the conversion of GLS to ITCs and making this biotransformation reliant on myrosinase-like activity of bacteria in the gut (92, 97, 123). Both *in vitro* and *in vivo* work has shown that the gut microbiota has myrosinase-like activity, converting GLS to ITCs, and select microbes shown to metabolize GLS is presented in Table 1 (99–102, 109, 125). Work conducted *in vitro* has shown direct relationships between specific gut microbes and ITC generation, typically through the use of microbial monocultures and purified GLS extracts (101, 102, 109). *Bacteroides thetaiotaomicron* has been shown extensively to possess myrosinase-like activity, both *in vitro* and *in vivo* (99, 124). *Lactobacillus agilis R16, Eneterococcus casseliflavus* CP1, and *Escherichia coli VL8, Escherichia coli* 1917 Nissile, have also been shown *in vitro* to possess myrosinase-like activity and produce ITCs from GLS including GTP, GER, GIB, and GRP (101, 102, 107, 109).

**Table 1:** Bacteria shown to metabolize glucosinolates (GLS).

**Bacteria**	**Effect on GLS**	**GLS studied**	**Model**	**References**
*Bacteroides thetaiotomicron*	Convert GLS to ITC	SNG, GRP	*In vitro*, monocolonized gonotbiotic mice	(99, 124)
*Bifidobacterium adolescentis*	Convert GLS to NITs	SNG	*In vitro*	(103)
*Bifidobacterium longum*	Convert GLS to NITs	SNG	*In vitro*	(103)
*Bifidobacterium psudoctenulatum*	Convert GLS to NITs	SNG	*In vitro*	(103)
*Enterococcus casseliflavus* CP1	Convert GLS to both NITs and ITCs	GER, GIB, GRP, GTP, SNG, GNT	*In vitro*	(101, 102)
*Enterococcus cloacae*	Convert GLS to NITs; Reduced GLS	GRP, GIB	*In vitro*	(110)
*Escherichia coli* 1917 Nissile	Convert GLS to NITs; Reduced GLS	GRP, GIB	*In vitro*	(110)
*Escherichia coli* VL8	Convert GLS to both ITC and NITs; Reduced GLS	GER, GIB, GRP, SNG, GTP, GNT	*In vitro*	(101, 102)
*Lactobacillus Agilis* R16	Convert GLS to both ITCs and NITs	SNG, GTP, GNT, GER	*In vitro*	(101, 102, 109)
*Lactobacillus plantarum* KW30	Convert GLS to NITs	GRP, GIB	*In vitro*	(110)
*Lactococcus lactis* KF147	Convert GLS to NITs	GRP, GIB	*In vitro*	(110)

*GER, Glucoerucin; GIB, Glucoiberin; GNT, Gluconasturtiin; GRP, Glucoraphanin; GTP, Glucotropaeolin; SNG, Singrin; ITC, Isothiocyanate; NIT, Nitrile*.

While these studies have generated a pool of microbes capable of myrosinase-like activity, their translation to human work is unclear. Many microbes found within the human gastrointestinal tract are unculturable, making their growth in a lab environment to conduct such studies a challenging feat. Additionally, the monocultures used in these studies fail to capture microbe-microbe interactions that occur in dynamic systems such as the gastrointestinal tract. Beyond these issues, the use of purified GLS does not capture the impacts of whole food matrix components on microbial communities, failing to address the most common way that GLS are received in the diet. Interestingly, many of these studies show high degradation of GLS yet low yield of ITCs and NITs and it is not clear if this is because of further metabolism of ITC and NIT, or metabolism of GLS to alternate endpoints (99–102, 109, 110, 126). This is complicated by the unstable nature of ITCs, which have been shown to spontaneously degrade and react with other compounds, and may lead to an incomplete understanding of conversion rate and yield of ITC from bacteria (127).

Clinical studies and animal models have provided further evidence that the gut microbiome can produce bioactive ITCs from GLS. Most studies investigating the role of the gut microbiota in GLS conversion utilize purified GLS extracts or whole cruciferous vegetables with heat-deactivated myrosinase, however, some use less conventional routes such as having participants swallow broccoli sprouts whole (65, 128–132). Work conducted in rodents and other animal models have additionally relied on gnotobiotic models to further characterize the role of the gut microbiome in GLS metabolism (100, 124, 133). Cumulatively, these studies have indicated that the gut microbiome is essential for conversion of GLS to ITCs in the absence of plant-derived myrosinase and have shown that without conversion to ITCs, GLS are biologically inert. Other studies using gnotobiotic animals inoculated with single strains of bacteria have demonstrated that biotransformation of allyl isothiocyanate from sinigrin *in vitro* does indeed translate to biotransformation *in vivo* (99). Curiously, some studies using humanized rodent models have failed to demonstrated conversion of GLS to ITCs while others have observed the generation of AITC from SNG in germ-free animals (99, 100). These discrepancies highlight the need for model systems which capture complex interactions and dynamic systems.

Clinical trials examining the role of the gut microbiota on the conversion of GLS to ITCs is far more limited at this time (62, 66, 131). These studies typically rely on the use of vegetables with heat deactivated-myrosinase and purified GLS extract to draw conclusions on the role of the gut microbiome (34, 35, 61, 65, 128, 130, 131). A study conducted by Fahey et al. identified large inter-individual variation in GLS metabolism and sought to explore the role of the human gut microbiota by utilizing broccoli sprout extracts (primarily containing GRP) where myrosinase was heat deactivated (66). One particularly noteworthy finding of this study was the general presence of four phenotypes of GRP metabolism in the population, high/fast, high/slow, low/fast, and low/slow converters; where high/low refers to conversion efficacy and fast/slow refers to if the bulk of the dose was converted within the first 8 h after consumption or between 8 and 24 h after consumption. These phenotypes may be related to differences in the subjects' microbiomes and the findings suggest that enterohepatic circulation and food-matrix effects could have a profound impact on microbial metabolism of GLS. Work conducted by Bheemreddy and Jeffrey has verified that GLS undergo enterohepatic circulation in rats, giving greater insight into systemic metabolism of both GLS and ITCs (133). This work complements earlier findings by Kassahun et al. who identified SFN-GSH and SFN-NAC in the bile of rats fed purified SFN (134). These findings are important as it suggests two different intervals in time when GLS metabolism occurs in the large intestine, where the majority of the gut microbiome resides. The first interval is metabolism of GLS directly following consumption when the GLS is not absorbed in the small intestine. The second time interval occurs when GLS are absorbed in the small intestine and go through enterohepatic circulation, returning as GLS in the gut where the factors influencing microbial metabolism (such as the food matrix, pH, and other compounds present) may be different from the first time interval.

Another landmark study on the role of the gut microbiome in GLS metabolism comes from Li et al. (60). Participants were fed cooked broccoli and ITC excretion in urine was measured over the course of 24 h. Fecal samples from the 10 highest and lowest ITC samples were then cultured and incubated again with purified GRP 1–2 months following the first feeding. This study found no significant differences in microbiome composition between high and low ITC excreters. Furthermore, *ex vivo* incubation of fecal samples from high and low ITC-producers resulted in non-significant differences in GLS degradation. While this is one of the only studies to directly measure relationships between the gut microbiota and GLS conversion to ITCs in humans, and has a sound study design, it has a few major limitations. At the time this study was conducted, bioinformatic technologies and software lacked modern power and taxonomical resolution. Additionally, in the fecal incubation studies, the researchers solely measured degradation of GLS as opposed to the metabolic products of GLS, failing to capture the entire picture. Nevertheless, the findings of this study also show the ephemeral nature of the gut microbiota and highlight that gut microbiome composition can change and thus may be targeted, through dietary or probiotics means.

The genes responsible for the microbial conversions of GLS to ITCs are still being elucidated. Three genes encoding β-glycosidase enzyme have been identified in *E. coli* strains (135). Two of these genes in particular, *bglA* and *ascB* were shown *in vitro* to degrade singrin to AITC (135). Another microbial myrosinase-like gene has been identified in *Citrobacter* isolated from soil, however, the relevance of this strain to the gut microbiome is unclear (136). A recent study has identified an operon in *Bacteroidetes thetaiotaomicron* that is responsive to GLS and is capable of metabolizing them to ITCs, and metagenomic analysis across multiple distinct populations suggested that this operon is widely distributed across the gut microbiome (124). Among *Bacteroidetes thetaiotaomicron* species, varying ability to convert GLS to ITCs was detected, suggesting that environment or horizontal gene transfer could influence the expression of GLS metabolizing gene (124). Furthermore, the bacterial GLS metabolizing genes had no identified homologs in *Lactobacillus agillis R16* and *E. coli VL8*, two other microbes known to metabolize GLS to ITCs, implying that multiple microbial metabolic pathways exist (101, 109, 124). Further work utilizing modern “omics”-level approaches will be necessary in identifying microbial genes, and their related enzymes, that are responsible for the conversion of GLS to ITCs as well as other microbial metabolites produced by GLS hydrolysis.

### GLS Hydrolysis to Biologically Inert Compounds

Beyond conversion to ITCs, GLS can be metabolized to biologically inert compounds, such as NITs (Table 1). Endogenous plant factors, such as ESP have been shown to skew GLS conversion by myrsosinase toward NIT production, as opposed to ITCs, however, ESP is deactivated under heat treatment (95, 96). Other factors, such as the presence of Fe^2+^ ions and acidic conditions can additionally lead to the production of NITs over ITCs, however, evidence *in vitro* has shown that regardless of pH microbes can produce NITs from GLS, suggesting distinct microbial metabolic pathways exist for this biotransformation (29, 31, 91, 101, 102, 137). Work conducted by Mullaney et al. has suggested that lactic acid bacteria, particularly *Lactobacillus plantarum* KW30 and *Lactococcus lactis* KF147 preferentially convert GRP and GIB to SFN-NIT and IBN-NIT, respectively, as opposed to ITCs (110). Other work has shown that some taxa, including *Enterococcus casseliflavus* CP1 and *Escherichia coli* VL8 are all capable of producing both NIT and ITC products from multiple GLS including SNG (Allyl-NIT, AITC), GTP (Benzyl-NIT, Benzyl-ITC), GNT (Phenethyl-NIT, Phenethyl-ITC), GER (ERN-NIT, ERN-ITC), GIB (IBN-NIT, IBN), and GRP (SFN-NIT, SFN) (101, 102). While these studies have contributed greatly to our understanding of microbial metabolism of GLS, they tend to rely heavily on monocultures and purified GLS extracts, failing to capture complex diet-microbe, microbe-microbe, and microbe-host interactions which are present in the human gut (101, 103, 107–110, 137).

While many microbes have been shown to convert GLS to NITs, the metabolic pathway underpinning this bioconversion still needs further elucidation. Work by Luang-In et al. has shown that bacterial β-O-glucosidase are capable of metabolizing desulfoglucosinolates, GLS without a sulfate group, to NITs (101, 138, 139). While desulfoglucosinolates exist naturally in plants, it is hypothesized that a bacterial sulfatase could fill the metabolic niche of converting GLS to desulfoglucosinolates. Desulfoglucosinolate hydrolysis results solely in the production of NITs and cannot result in ITC production. *In vitro* work has identified two microbial enzymes responsible for this conversion, bgl4 and Tp8, which have been shown to yield ~15 and ~70% NITs from desulfoglucosinolates, respectively (138, 139). The large difference in yields from these two enzymes is most likely due to differences in structure and origin. Work by Lu et al. searched for the bacterial sulfatase present, and while detecting one, they found it had low affinity for SNG concluding that it was not the enzyme responsible for the biotransformation (140). Additionally, a novel bacterial sulfatase was discovered in members of bacterial family Clostridiaceae which may be responsible for the conversion of GLS to desulfoglucosinolates (141). Coinciding with this hypothesis, a recent study by Kaczmarek et al. found that broccoli consumption lead to a decrease in abundance of bacteria from family Clostridiaceae (142). Bacteria from the Clostridiaceae family were also found to be negatively correlated with the maximum peak of GLS metabolites in plasma (142). A microbial enzyme directly responsible for the desulfation of GLS to desulfoglucosinolates has not yet been detected, thus, the impact on this desulfation *in vivo* is not yet known. Individuals with gut microbiomes enriched with bacteria capable of converting GLS to desulfoglucosinolates could result in lower bioavailability of ITC from a dose of GLS. Future work is needed to identify microbial enzymes responsible for the desulfation of GLS by gut microbes and to determine the impact of these bioconversions on efficacy of broccoli sprout supplementation.

### GLS Redox Reactions

Beyond hydrolysis, the gut microbiota has been shown to utilize redox reactions to biotransform not only GLS but also NITs. More specifically, *in vitro* experiments with bacteria have shown that many microbes are capable of reducing GLS such as GIB and GRP to their redox partners glucoiberverin and GER, respectively (110). The conversion of SFN to ERN has been observed *in vivo*, both in human and animal studies (34, 62, 101, 102, 132, 133, 143). *Enterobacter cloacae* ATCC13047, *Escherichia coli* 1917 Nissile, and *Escherichia coli* VL8 have all been shown to reduce GRP and GIB as well as the nitriles IBN-NIT and SFN-NIT, *in vitro* (102, 110). In human studies, high levels of inter-individual variation in conversion of SFN to ERN was observed and it is still unclear if this biotransformation occurred before or after the hydrolysis of GLS to ITC and if the reduction occurred by endogenous host enzymes or in the gut lumen by the gut microbiome (143). Reduction of SFN-NIT to ERN-NIT and IBN-NIT to iberverin-NIT was also observed in fecal cultures *in vitro*, however, it has yet to be shown if this occurs in humans (110). Bacterial methionine sulfoxide reductase A is a prime candidate to reduce ITCs, like SFN to ERN, because it is found in all aerobic organisms and thus may alter ITC metabolism as they travel through the gut (144, 145). Microbes shown to complete redox reactions on GLS are shown in Table 1.

The implications of these redox-reactions are not well-known in humans. In studies solely examining the bioavailability and conversion of a single GLS to ITCs (e.g., GRP to SFN), these conversions can lead to a decrease in apparent bioavailability or increased variability between subjects due to differing levels of conversion. The impact of redox reactions on the bioactivity of ITCs also needs further investigation. ERN is shown to possess similar bioactivity as its redox partner SFN, however, its parent GLS, GER, has been shown to possess direct antioxidant properties and ERN is also believed to possess these properties (19–21, 41). Interestingly, when GER reacts with hydroperoxides it becomes oxidized to GRP, the precursor of SFN (41). Further investigation is needed to identify the implications of GLS, ITC, and NIT redox transformations *in vivo*.

## Cruciferous Vegetables and Gut Microbiome Composition

### Impacts of Cruciferous Vegetable Consumption on Microbiome Structure

Compounds from cruciferous vegetables have been shown to modulate the gut microbiome, altering its structure and potentially metabolic function, having implications in both cruciferous vegetable metabolism and general health (104, 105, 146–148). This implies a direct relationship between the composition of the gut microbiome and the metabolism of GLS, specifically the generation of ITCs. Clinical trials have shown that consumption of a diet rich in cruciferous vegetables, compared to a cruciferous vegetable devoid diet, significantly alters the composition of the gut microbiome (146). Interestingly, each individual responded uniquely to cruciferous vegetable consumption, suggesting that basal microbiome composition may impact outcome. A similar shift in microbiome composition was observed in rats in response to cruciferous vegetable consumption (105). In rodents, bacteria from the phylum Verrucomicrobia and from the species *Akkermansia municiphila* have been shown to increase with consumption of broccoli while the genus *Lactobacillus* has been shown to decrease with broccoli consumption (105, 148). Conversely, bacteria from genus *Oscillibacter, Ethanoligenens*, and *Gordonibacter* have been shown to increase with broccoli consumption (105, 147). In humans, *Eubacterium hallili, Phascolarctobacterium faecium, Aliestipes petrudeinis*, and the genus *Eggerthella*. were found to decrease with cruciferous vegetable consumption (60, 146). In humans, the family Desulfovibrionacaeae was found to increase with broccoli sprout consumption while in rats this family was decreased with consumption (60, 104). The differential changes in Desulfovibrionacaeae across studies could be due to differences in host factors, considering that one study was conducted in rats while the other was conducted in humans, or the source of vegetable with the Wu et al. utilizing freeze dried raw broccoli while the Li et al. used fresh broccoli sprouts. Similar discordant changes were observed in bacteria from the genus *Alistipes* in multiple studies conducted in humans (60, 146, 149). Factors driving these differences could stem from differences in study populations as one was conducted in the US and the other in the UK or differences in the foods consumed alongside the cruciferous vegetables.

A recent study conducted in humans found that consumption of cooked broccoli and raw daikon radish resulted in an increase bacteria from the genera *Bacteroides* and phylum Bacteroidetes and a decrease in bacteria from the phylum Firmicutes, compared to control (142). Another study found that consumption of a diet high in cruciferous vegetables lead to a decrease in sulfate-reducing bacteria compared to a diet low in cruciferous vegetables, specifically bacteria from the order Clostridiales (149). A list of bacterial genera altered by cruciferous vegetable consumption can be found in Table 2. This list focuses on studies completed in healthy individuals and animal models, however, work has also been conducted in disease models (150–153).

**Table 2:** Bacteria significantly altered with cruciferous vegetable consumption.

Bacteria^*^	**Δ With consumption**	**Model**	**References**
*Akkermansia*	Increase	Rat	(105, 148)
*Alistipes*	Decrease	Rat	(147)
	Increase	Human	(146)
	Increase	Human	(60)
	Decrease	Human	(149)
*Bacteroides*	Increase	Human	(142)
Phylum: Bacteroidetes	Increase	Human	(142)
*Blautia*	Decrease	Rat	(105)
*Burkholderiales*	Decrease	Human	(146)
*Clostridium*	Increase/decrease	Rat	(105)
	Decrease	Human	(149)
*Coprococcus*	Decrease	Rat	(105)
*Dehalobacterium*	Decrease	Human	(149)
*Desulfovibrio*	Increase	Human	(60)
	Decrease	Mice	(104)
*Dorea*	Decrease	Rat	(105)
*Eggerthella*	Increase	Human	(146)
*Ethanoligenens*	Increase	Rat	(147)
*Eubacterium hallii*	Increase	Human	(146)
Phylum: Firmicutes	Decrease	Human	(142)
	Decrease	Human	(149)
Family: Lachnospiraceae	Decrease	Rat	(105)
	Increase	Mice	(104)
Family: Rikenellaceae	Decrease	Human	(149)
Family*:* Ruminococcaceae	Increase	Rat	(105)
	Decrease	Human	(149)
Family: S24-7	Increase	Rat	(105)
*Gordinobacter*	Increase	Rat	(147)
*Lactobacillus*	Decease	Mice	(148)
*Lactococcus*	Decrease	Rat	(147)
	Increase	Human	(60)
*Lutispora*	Decrease	Rat	(147)
Family: Mogibacteriaceae	Decrease	Human	(149)
*Mucispirillum schaedleri*	Decrease	Mice	(148)
*Oscillobacter*	Increase	Rat	(147)
*Oscillospira*	Increase	Rat	(105)
*Papillibacter*	Decrease	Rat	(147)
*Prevotella*	Increase	Rat	(105)
*Rc4-4*	Increase	Rat	(105)
*Streptococcus*	Decrease	Rat	(147)
*Tannerella*	Decrease	Rat	(147)
*Vampirovibrio*	Decrease	Rat	(147)

* *Genera unless otherwise stated*.

Cruciferous vegetable induced changes in the microbiome may have a functional impact on the microbiome and in turn influence the host. This was recently highlighted in a paper which showed that changes induced by consumption of cooked broccoli and raw daikon radish were predicted to be associated with changes in microbial genes involving the endocrine system, transport and catabolism, and energy metabolism (142). Due to predictive nature of these analyses, future studies examining functional alterations to the gut microbiome following cruciferous vegetable consumption would benefit from the use of metagenomic methods and the integration of metabolomics data to further address how the changes in bacteria may influence the host. Kellingray et al. found that broccoli consumption lead to a decrease in sulfate-reducing bacteria, which are associated with gastrointestinal disorders such as ulcerative colitis and irritable bowel syndrome (149). Furthermore, sulfate is a product of GLS conversion to desufloglucosinolates, thus, the reduction in sulfate-reducing bacteria could potentially contribute to the increased production of ITCs by decreasing GLS conversion to desufloglucosinolates. Unfortunately, Kellingray and colleagues did not measure GLS, NIT, nor ITC metabolites so additional experiments are needed to test this hypothesis. Cumulatively, these findings suggest that cruciferous vegetable consumption could not only impact host health through the generation of ITCs and other bioactives, but also by altering overall gut microbiome composition and metabolism and thus host health.

While it has been well-established that consumption of broccoli sprouts significantly alters the composition of the gut microbiome, the specific compounds within broccoli that are responsible for this change are still unclear. Initial hypotheses pointed toward GLS, and their downstream metabolites ITCs, as responsible for the microbiome-modulatory effect of cruciferous vegetables due to the anti-microbial nature of ITCs (154–157). In contrast, recent evidence has suggested that broccoli itself is responsible for the changes (104). Two separate feeding studies in rodents found that hydrolyzed broccoli, where all GLS had been converted to ITCs, and broccoli containing intact GLS, lead to changes in gut microbiome composition while GLS supplementation, in the absence of broccoli, did not change microbiome composition (104, 105). Interestingly, in both studies treatment with whole broccoli, regardless of GLS hydrolysis, resulted in similar microbial community composition while GLS supplementation alone did not result in change relative to a broccoli-free control (104, 105). Taken together, these results suggest that while the non-GLS components of broccoli are responsible for the microbiome-modulatory properties of these foods, ITCs could act synergistically with these compounds.

### Alterations to Microbial Metabolism of Cruciferous Vegetables With Prolonged Exposure

Alterations to the gut microbiome through long-term consumption of cruciferous vegetables also has been observed to impact GLS metabolism. Rodent studies have reported an increased myrosinase-like activity by gut microflora following prolonged consumption of broccoli sprouts, typically 14 days or longer (104, 105). In humans, one study examining the bioavailability of SFN from a GRP rich powder over the course of 84 days found a gradual increase in SFN bioavailability over the course of the study (131). The authors of this study did not examine the gut microbiomes of their participants, but they speculate the increase in SFN bioavailability could be driven by changes in the composition of the gut microbiome. Liu et al., found that consumption of both cooked broccoli and purified GRP, lead to an increase in myrosinase-like activity in the gut microbiome (105). Conversely, the Wu et al. study found that raw broccoli lead to an increase in myrosinase-like activity while administration of SNG, the GLS precursor to AITC, did not (104). A possible explanation for the increase in myrosinase-like activity from GLS exposure could be due to induction of GLS-metabolizing genes through the operon first identified by Liou et al. in *Bacteroides thetaiotaomicron* (124). Overall, consumption of cruciferous vegetables can alter not only gut microbiome composition, but also gut microbiome metabolism. Further investigation is needed to understand these alterations in humans, as well as their impact on not only GLS metabolism but overall human health.

## Discussion—Metabolomics and Microbiome Analysis for the Future

To address gaps in knowledge in the role of the microbiome in driving inter-individual variation in GLS metabolism, a systems-biology approach leveraging recent technological advances can be utilized. High throughput sequencing (HTS) allows rapid and inexpensive deep sequencing of microbial samples, and improvements in bioinformatic technology have led to an increase in our power to analyze data (158, 159). These technological and methodological advancements have resulted in an abundance of studies utilizing 16S sequencing, revealing the composition of the gut microbiome. These types of studies will allow researchers to detect alterations to gut microbiome composition with cruciferous vegetables and identify taxa driving GLS metabolism *in vivo*. While these studies expand our knowledge of the gut microbiome, they lack the ability to tell us the functional role of these microbes. In contrast to 16S sequencing, metagenomic sequencing allows for identification of microbial genes enriched by experimental conditions, giving explanation of both *which* microbes are present and *what* they do (160). Despite its power, metagenomic sequencing has major limitations, specifically in the identification of novel genes responsible for microbial metabolism. Gene function is typically determined by comparing sequences against homologous genes, however, if homologous genes do not exist within annotated databases the functional aspects of metagenomic sequencing can fail to be captured (160). Nevertheless, metagenomics offers the potential to understand which metabolic niches GLS-metabolizing microbes fill, and determine the implications of these microbes not only on GLS metabolism, but more broadly on human health.

New methods have been proposed to bridge the gap between taxonomy and function, chiefly through the use of multi-omic integration to address the shortcomings of metagenomic sequencing (161). An example of a multi-omic approach would be to combine metabolomics and 16S sequencing data to uncover the influence of the microbiome in metabolite generation. Multi-omic methods can include data-driven approaches, which use an untargeted approach to analyze data and extract features of interest. Additionally, knowledge-based approaches use prior knowledge to find relationships between features. Data-driven approaches are typically based on statistical and machine learning techniques such as network analysis, regression, decision trees, and data reduction techniques like principal component analysis (regularized), canonical correlation analysis, and partial least squares (162–164). Knowledge-based approaches leverage databases and use techniques such as set-based enrich analysis, pathway analysis, and constraint-based metabolic modeling (162). All these methods rely on the integration of multiple omics technology, chiefly metatranscriptomics, metaproteomics, and metabolomics which capture perturbations to the gut microbiome at a more precise level than metagenomics alone (161).

Particularly relevant to GLS metabolism will be the integration of metabolomics data with microbiome data [reviewed in (165)] which could identify associations between members of the gut microbiome and specific microbial metabolites generating novel hypotheses for more targeted investigation. For example, untargeted metabolomics and 16S sequencing methods may shed light on why many of the *in vitro* studies of bacterial metabolism found up to 100% GLS degradation by bacteria, while a significant portion of the ITC (or related metabolites) products were unaccounted for (101, 102, 110, 140). The presence of unknown microbial metabolites may be a contributing factor and this multi-omic approach can capture not only ITCs, NITs, and their conjugates, but other products of microbial cruciferous vegetable metabolism. Through these approaches novel bioactives may also be discovered, further uncovering components of cruciferous vegetables which are responsible for their microbiome-modulatory effects as well as their efficacy in cancer prevention.

Metabolomics on human plasma coupled with bacterial sequencing following broccoli consumption can be also be used to find microbes associated with circulating SFN and SFN-NIT levels. Metabolomics captures host metabolites, as well as microbial metabolites, giving greater insight into inter-individual variation. While a large portion of both the human and microbial metabolome are unannotated, advances in computational mass spectrometry have helped overcome this barrier through the use of machine learning techniques for MS/MS matching and tentative metabolite annotation (166–168). Future studies utilizing multi-omic approaches will not only aid in identifying factors driving inter-individual variation in GLS metabolism, but also may lay the groundwork for how an individual's microbiome could be altered to improve ITC bioavailability from food and thus, may lead to improvements in ITC bioavailability and affect efficacy in cancer prevention.

## Conclusions

Growing evidence points toward the gut microbiome as an important player in determining ITC bioavailability in humans following GLS consumption. It is clear from *in vitro* studies that members of the gut microbiome can metabolize GLS to ITCs (*Lactobacillus agilis* R16*, Enterococcus casseliflavus* CP1, and *Escherichia coli* VL8) and NIT (Lactic Acid Bacteria). Thus, understanding the role of the gut microbiome in ITC production is paramount to the use of cruciferous vegetables as a cancer prevention strategy, as changes to GLS metabolism by the gut microbiome may lead to the decrease of cancer-fighting phytochemicals such as SFN, and increase the production of biologically inert ones like SFN-NIT. Consumption of cruciferous vegetables has additionally been shown to alter the composition of the gut microbiome, not only shifting its structure, but also its metabolic abilities toward ITC production. Understanding the role of the gut microbiome in the metabolism of GLS, specifically their conversion to ITC, is important to understanding the drivers of inter-individual variation in humans. Without addressing the factors that drive the high variability in ITC absorption and excretion observed in human clinical trials, translating the chemopreventative properties of cruciferous vegetables from the lab bench to the clinic is a challenge. Future studies should utilize multi-omics approaches to better understand the interplay between GLS metabolism, the gut microbiome and cancer prevention.

## Author Contributions

JB, LB, and EH have been responsible for the conception of this article and drafting the manuscript. JB created the figure and tables. LB and EH mentored and critically reviewed this work. All authors read and approved the final manuscript.

## Funding

This work was supported by United States Department of Agriculture National Institute of Food and Agriculture (NIFA-2020-67001-31214) to EH, National Institute of Health P30 ES03028, as well as funding from Oregon Agricultural Experimental Station (W4002; OR00735).

## Conflict of Interest

The authors declare that the research was conducted in the absence of any commercial or financial relationships that could be construed as a potential conflict of interest.

## Publisher's Note

All claims expressed in this article are solely those of the authors and do not necessarily represent those of their affiliated organizations, or those of the publisher, the editors and the reviewers. Any product that may be evaluated in this article, or claim that may be made by its manufacturer, is not guaranteed or endorsed by the publisher.

## Abbreviations

AITC: Allyl Isothiocyanate
ERN: Erucin
GER: Glucoerucin
GIB: Glucoiberin
GLS: Glucosinolate
GNP: Gluconastrurtiin
GRP: Glucoraphanin
GSH: Glutathione
GST: Glutathione-S-Transferase
GTP: Glucotropaeolin
IBN: Iberin
ITC: Isothiocyanate
NAC: N-Acetyl Cysteine
NIT: Nitrile
SFN: Sulforaphane
SNG: Singrin.
